# Norwegian midwives' perceptions of their practice environment: A mixed methods study

**DOI:** 10.1002/nop2.358

**Published:** 2019-08-06

**Authors:** Mirjam Lukasse, Lena Henriksen

**Affiliations:** ^1^ Institute of Nursing and Health Promotion, Faculty of Health Sciences Oslo Metropolitan University Oslo Norway; ^2^ Division of General Gynaecology and Obstetrics Oslo University Hospital Oslo Norway

## Abstract

**Aim:**

To investigate Norwegian midwives’ perceptions of their working environment.

**Design:**

A nationwide postal survey in 2014 collected information from 489 midwives, including the Practice Environment Scale and seven open‐ended questions concerning the workplace.

**Methods:**

Psychometric‐, descriptive‐ and comparative analysis was used for the quantitative data and content analysis for the qualitative data.

**Results:**

Psychometric analyses yielded five subscales: *Quality of management; Resource adequacy; Midwife‐doctor relations; Opportunities for development;* and *Midwifery foundation for care*. Content analyses identified four main themes: *Lack of resources; Insufficient support; Staying in midwifery;* and *Lack of influence*. Subthemes only found in the qualitative analysis were as follows: *Fear of adverse events* and *The strain of shift work*. Most midwives rated the PES subscales *Midwife‐doctor relations* and *Quality of management* favourable. In contrast, the theme *Lack of influence* showed that midwives felt powerless in a constantly changing work environment and ruled by the medical model of care.

## 1 INTRODUCTION

Midwives have always had challenging working conditions, working shifts or on‐calls and weekends, as well as experiencing peaks in the workload that no duty roster can prepare for (Mollart, Skinner, Newing, & Foureur, 2013; Pezaro, Clyne, Turner, Fulton, & Gerada, 2016; Yoshida & Sandall, 2013). Midwifery work can in addition be described as intense, emotionally demanding, with the risk of experiencing traumatic events (Coldridge & Davies, 2017; Hunter, 2001; Pezaro et al., 2016; Wahlberg et al., 2017).

In high‐income countries, the past decades have seen statistically significant changes in the work‐content and environment of midwives. The increase in demands for cost‐effectiveness has led to the reduction of hospital beds resulting in shorter hospital stay (Brown, Small, Faber, Krastev, & Davis, 2002). More technical equipment is introduced, while midwives appear to have less time to be ‘with the woman’ (McCool & Simeone, 2002; Zwelling, 2008). There is an increased awareness of the price tag attached to all ‘items’ used in care, including midwives. The structure and organization of the workplace has an influence on the well‐being of midwives as studies on burnout and empowerment of midwives show (Fenwick, Lubomski, Creedy, & Sidebotham, 2018; Fenwick, Sidebotham, Gamble, & Creedy, 2018; Lukasse & Pajalic, 2016). Thus, an investigation of how midwives perceive their work environment is important.

## 2 BACKGROUND

Internationally, the midwifery workforce is ageing and facing diminishing participation (Hildingsson & Fenwick, 2015; Pugh, Twigg, Martin, & Rai, 2013). Midwives leave the workforce because of unsuitable hours, increasing workload, insufficient clinical support and inadequate education and professional development opportunities (Hildingsson & Fenwick, 2015; Kirkham, 2007). Opportunities to influence practice and decision‐making, feeling supported by colleagues and managers, adequate resources and close relationships with clients have been identified as factors that encourage midwives to stay (Sullivan, Lock, & Homer, 2011).

In Norway, midwifery is a specialization after nursing (Lukasse, Lilleengen, Fylkesnes, & Henriksen, 2017). Midwives work in close collaboration with obstetricians and general practitioners. Almost all births take place in hospital, while routine antenatal care, except for routine ultrasound, is provided in the community (Blix, Huitfeldt, Oian, Straume, & Kumle, 2012). Much of the work midwives do, especially in hospital, is increasingly directed (guided/dictated) by national and local procedures, leaving individual midwives with diminishing professional autonomy. Doctors are the common authors of procedures and midwives have limited influence over them. Thus, while doctors are not usually present at straightforward uncomplicated births, the medical profession instructs midwives how to conduct them. First‐line managers for midwives are usually other midwives. While higher up in the hierarchy most leadership positions are held by medical doctors.

The past decades have seen a centralization of care with the closure of many small maternity units and an increase in number of births in the already large units throughout Norway (Huitfeldt, Voldner, & Blix, 2016; Nilsen, Daltveit, & Irgens, 2001). Norway has few midwifery‐led units, there are few independent midwives and planned home births are rare (Huitfeldt et al., 2016). Midwives working in hospitals and midwife‐led units are required to work shifts and weekends. Community midwives and those working in outpatient departments, for example with routine ultrasound, work office hours and weekdays only.

The Practice Environment Scale (PES) was originally developed by Lake in 2002 to assess which aspects were important in nurses’ perceptions of the quality of their working environment (Lake, 2002). Since then, the use of the scale has grown across different clinical settings and countries (Warshawsky & Havens, 2011). The 31‐item PES was adapted for use with midwives by Pallant, Dixon, Sidebotham, and Fenwick (2016) by removing item 31 and changing the perspective from nursing to midwifery. Pallant et al. (2016) found that all the subscales resulting from their factor analysis were significant predictors of considering leaving the profession within the last 6 months. In contrast to some other nations, leaving the profession has not been a concern in Norway. However, Norway has an ageing midwifery workforce and recruiting motivated midwives is important. How the current midwives perceive their practice environment plays a role in attracting new midwives. The research question for this study was as follows: ‘How do Norwegian midwives perceive their practice environment?’

To answer this question, we conducted the following steps. Firstly, we assessed the psychometric properties of the PES and adapted it to the Norwegian midwifery setting. Secondly, we explored factors associated with an unfavourable working environment. Thirdly, we analysed the responses to the open‐ended questions on midwives’ working environment. Finally, we interpreted the results from the PES and the open‐ended questions jointly.

## 3 THE STUDY

### 3.1 Design

A postal survey study was designed to investigate midwives’ working situation and emotional well‐being. Almost identical studies have been performed in Australia, New Zealand and Sweden (Hildingsson, Westlund, & Wiklund, 2013; Jordan, Fenwick, Slavin, Sidebotham, & Gamble, 2013; Pallant, Dixon, Sidebotham, & Fenwick, 2015). These and our study are part of a growing international network called WHELM, the Work Health and Emotional Lives of Midwives network. The questionnaire was translated from English to Norwegian by a professional translator and checked against the Swedish (very close to Norwegian) and assessed for face validity by two midwives.

### 3.2 Sample

In September 2014, questionnaires, together with a response envelope, were sent to a random sample of 1,500 midwives registered with either one of the two midwifery unions in Norway. The two unions together organize ~99% of all active midwives in Norway. Most midwives are organized in the Norwegian Association of Midwives (Den norske jordmorforening), while the rest are organized in the midwifery group of the Norwegian Nurses Organization (Jordmorforbundet). The sampling method ensured proportional sampling from both organizations. The number of midwives in active midwifery practice was around 3,000 at the time of the study. A third party performed the random sampling and posting of the questionnaires. No reminder was sent as the questionnaire was totally anonymous and the researchers knew neither whom they were sent to, nor who responded.

Of the 1,500 questionnaires, 1,458 were eligible after exclusion of 26 due to wrong address (moved, unknown) and 16 midwives who no longer worked in midwifery. Of the 1,458 eligible, 598 (41%) completed the questionnaire. To compare with the results by Pallant et al. (2016), we included only midwives who indicated that they were, at least part‐time, employed in a hospital setting (*N* = 496). In addition, we removed seven midwives from the dataset as they had more than 10 of the 30 items of the PES missing. Thus, the quantitative dataset consisted of 489 midwives.

### 3.3 Data collection

The questionnaire consisted of four parts. The first part collected background demographic information, such as age, marital status, main and secondary area(s) of practice, years of experience, current post, type of midwifery education and other education.

The second part of the questionnaire inquired into midwives’ health and well‐being. The third part of the questionnaire included the Perceptions of Empowerment in Midwifery Scale and the Practice Environment Scale. The paper by Pallant et al. (2016) presenting the ‘PES:Midwives’ had not been published by the time we performed our study. Therefore, we used the 30 items adapted to midwifery by Pallant et al. (2016), which were the exact same items used in the Swedish study (Hildingsson & Fenwick, 2015). The final and fourth part of the questionnaire consisted of 7 open‐ended questions, allowing 2,5 pages (size A4) for answers, concerning the working environment and midwives’ experiences at/of work (Table S1).

## 4 METHOD FOR THE QUANTITATIVE DATA ANALYSIS

### 4.1 Statistical analysis

There were few missing data after the exclusion of participants with >10 items missing. Missing data were not replaced. Principal component analysis (PCA) was conducted using SPSS version 22 to explore the underlying structure of the 30 PES‐Midwives items in the Norwegian setting. To assess suitability of the dataset for PCA, the Kaiser–Meyer–Olkin test of sampling adequacy (values above 0.6) and Bartlett's test of sphericity (*p* < .001) were conducted. Our selection of factors was guided by eigenvalues above 1, Cattell's scree test and parallel analyses. Parallel analyses compare the eigenvalues from the exploratory factor analyses for PES with those obtained from a randomly generated data file of the same size. Only factors with eigenvalues exceeding the corresponding eigenvalue of the random dataset were kept. Parallel analysis was performed using the software developed by Watkins.

The solution was rotated using Oblimin rotation to assist interpretation, with items being considered for removal from the scale if they failed to load above 0.4 on any factor, or if they showed substantial cross loadings on two or more factors. Preliminary analyses were conducted to confirm suitability of the dataset for factor analysis. The Kaiser–Meyer–Olkin measure of sampling adequacy of 0.906 and the significant Bartlett's test of sphericity (*p* < .001) supported its factorability. Principal component analysis revealed 6 factors with eigenvalues above 1., explaining 57. 8% of the variance. The scree plot suggested a 4‐factor solution, while the parallel analyses suggested that no more than five factors should be retained for further investigation. The sixth factor was smaller than that obtained from a random dataset of the same size and therefore not be considered reliable.

Principal component analysis (PCA) of the five‐component solution with Oblimin rotation initially explained 54.0% of the variance. We subsequently removed 11 items, due to low communality values (below 0.4) or substantial cross loading on more than two factors.

The items of each of the 5 factors (subscales) in the final solution were summarized and divided by the number of items to create a sub‐score. Principal component analysis with three and four factors was explored but difficult to interpret and therefore rejected.

The subscales were in addition divided into unfavourable (mean subscale score <2.5) and favourable (mean subscale score ≥2.5) (Hildingsson & Fenwick, 2015; Pallant et al., 2016). Internal consistency reliability for the subscales was calculated using Cronbach's alpha coefficients. Descriptive analysis was performed for each of the subscales. Cross‐tabulation and Pearson's chi‐squared test were used to study differences in midwives’ characteristics and an unfavourable working environment (<2.5 score) for each of the subscales. Logistic regression analysis was performed for all the 5 factors entering all the characteristics from the first analyses using backward stepwise conditional modelling keeping only statistically significant characteristics. All analyses were two sided at α = 0.05. Statistical package SPSS version 22 was used to conduct all analyses.

### 4.2 Method for the qualitative data analysis

We used content analysis as described by Graneheim and Lundman, to analyse the qualitative data (Graneheim & Lundman, 2004). The length of the texts varied from half a page to 2,5 pages. The analysis was performed using the following steps: (a) a thorough review of the all‐text from the open‐ended answers to become familiar with the data; (b) division of all the written texts into meaning units; (c) condensing the meaning units and labelling them with codes; (d) distributing the codes into categories; (e) abstracting and condensing the categories into subthemes; and (f) analysing the subthemes and unifying them into four main themes and one overall theme. To strengthen trustworthiness, both authors throughout the analysis process discussed the codes, subthemes and themes until agreement was reached. Examples of analysis with meaning units, codes, subthemes and theme are given as supporting information, Table S2. Quotations were chosen to illustrate the themes and subthemes.

### 4.3 Data integration

Integration of both quantitative and qualitative data is cited as the essence of mixed methods research (Johnson & Onwuegbuzie, 2004). Both datasets were analysed independently, each author with the responsibility for their analyses. The authors were in dialogue when analysing and interpreting, to give both datasets equal status and gain information on the overall main aim of the study that is to investigate midwives’ perceptions of their practice environment.

## 5 RESULTS

### 5.1 Quantitative

The demographic characteristics of the participating midwives are presented in Table 1. Principal component analyses of the 30‐item PES in our Norwegian sample resulted in a solution using 19 items (questions) which explained 66.2% of the variance with 5 factors with an eigenvalue above 1 (Table 1). The factors (subscales) were labelled: *Quality of management; Resource adequacy; Midwife–doctor relations; Opportunities for development;* and *Midwifery foundation for care* (Table 2). Correlation between the factors ranged from 0.205–0.559 suggesting that they should not be combined into a total score (Table 3). Cronbach's alpha for 3 of the 5 scales was above 0.8 (good), one was above 0.7 (acceptable) while one was 0.683 (slightly low) and probably due to few items in that scale (Pallant, 2013; Table 3).

**Table 1:** Characteristics of the sample, *N* = 489

Characteristics	Categories within the characteristics	*N* (%)
Age	<40 years	138 (28.2)
	≥40 years	351 (71.8)
Main area of practice	Community A/N care	29 (5.9)
	Hospital ward	361 (73.8)
	Normal birth unit	21 (4.3)
	Outpatients Department	41 (8.4)
	Education, management and other	37 (7.6)
Type of post	Midwife without leadership/specialist duties	387 (79.2)
	Midwife with leadership/specialist duties	99 (20.8)
Content in practice	One area	243 (49.7)
	More than one area	241 (50.3)
Size maternity unit	<2,500 births per year	265 (54.2)
	≥2,500 births per year	223 (45.8)
Working hours	Full time	220 (45.0)
	Part‐time	267 (55.0)
Work distribution	Daytime, weekdays only	66 (14.0)
	Shifts/weekends	421 (86.0)
Midwifery experience	<10 years	168 (34.4)
	≥10 years	319 (65.6)
Academic degree	None	252 (51.5)
	Any	237 (48.5)
Recent organization change	No	279 (57.0)
	Yes	171 (43.0)

**Table 2:** Pattern matrix of the five‐component solution Principal Component Analysis with Oblimin rotation

	Factor 1	Factor 2	Factor 3	Factor 4	Factor 5
% Variance explained by each factor	33.0%	11.3%	10.1%	6.4%	5.5%
Factor 1: Quality of management					
10. A Midwifery Unit Manager who is a good manager and leader	**0.921**	−0.053	−0.032	−0.015	−0.008
3. A Midwifery Unit Manager that is supportive	**0.896**	−0.049	−0.034	0.012	−0.053
28 Midwife Managers consult with staff on daily problems and procedures	**0.771**	0.003	−0.044	−0.058	0.139
21. Hospital management that listens and responds to employee concerns	**0.619**	0.218	0.111	0.103	−0.104
13. Praise and recognition for a job well done	**0.580**	0.067	0.055	0.131	0.068
20. A midwifery unit manager who backs up the nursing staff in decision‐making even if the conflict is with a doctor	**0.539**	0.012	0.175	0.067	0.097
Factor 2: Resource adequacy					
9. Enough midwives to provide quality patient care	−0.007	**0.901**	0.019	−0.030	−0.006
12. Enough staff to get the work done	0.001	**0.885**	0.038	0.009	−0.088
1. Adequate support services allow me to spend time with my clients	−0.069	**0.730**	−0.091	0.179	0.021
8. Enough time and opportunity to discuss client care with other midwives	0.129	**0.648**	0.002	−0.147	0.160
Factor 3: Midwife‐doctor relations					
16. Good teamwork between midwives and doctors	−0.002	−0.024	**0.887**	0.003	0.026
2. Doctors and midwives have good working relations.	−0.052	−0.028	**0.877**	0.012	0.024
24. Collaboration (joint practice) between midwives and doctors	0.109	0.013	**0.753**	0.045	0.005
Factor 4: Opportunities for development					
5. Career development/clinical ladder opportunity	0.039	−0.042	0.004	**0.856**	0.034
17. Opportunities for advancement	−0.067	0.002	0.049	**0.796**	0.025
4. Active staff development or continuing education programme for midwives	0.167	0.095	0.004	**0.700**	0.18
Factor 5: Midwifery foundation for care					
26. Midwifery care is based on a midwifery model rather than a medical model	−0.113	0.082	0.142	−0.053	**0.817**
18. A clear philosophy of midwifery that pervades the patient care environment	0.116	0.045	0.118	0.051	**0.665**
29. Written up‐to‐date care pathways for all women	0.131	−0.058	−0.160	0.142	**0.625**

Notes

The items are collected within the given factors based on the bold values.

**Table 3:** Inter‐correlations and descriptive statistics of the PES subscales, *N* = 489

	Quality of management	Resource adequacy	Midwife‐doctor relations	Opportunities for development	Midwifery foundation for care
Mean (*SD*)	2.71 (0.58)	2.38 (0.58)	3.00 (0.49)	2.20 (0.83)	2.37 (0.54)
Proportion of midwives with <2.5 score	28.8%	51.1%	11.5%	71.6%	59.3%
Internal Reliability (Cronbach's alpha)	0.869	0.829	0.821	0.775	0.683
Mean inter‐item correlation	0.523	0.544	0.605	0.533	0.371
Correlations among the subscales (r)					
Quality of management	–	0.338	0.325	0.559	0.534
Resource adequacy	0.338	–	0.205	0.280	0.281
Midwife‐doctor relations	0.325	0.205	–	0.239	0.369
Opportunities for development	0.559	0.280	0.239	–	0.430
Midwifery foundation for care	0.534	0.281	0.369	0.430	–

Midwives on average evaluated the factors: *Resource adequacy, Opportunities for development; and Midwifery foundation for care* as unfavourable, while the factors *Quality of management* and *Midwife‐doctor relations* were generally evaluated favourably (Table 3). Midwives aged less than 40 years of age were significantly more likely to have an unfavourable evaluation of the factors: *Resource adequacy; Opportunities for development;* and a *Midwifery foundation for care* (Table 4). However, this association lost its significance when entered in the multivariate regression analyses (Table 4). Working at a normal birth unit was associated with an unfavourable rating of: *Quality of management; Resource adequacy;* and *Opportunities for development* (Table 4). This association remained in the multivariate regression analyses (Table 5).

**Table 4:** Proportion of unfavourable subscale scores (mean < 2.5) by midwives’ characteristics (row percentages)

Characteristics	Quality of management	Resource adequacy	Midwife–doctor relations	Opportunities for development	Midwifery foundation for care
	*N* (%)	*N* (%)	*N* (%)	*N* (%)	*N* (%)
Age					
<40 years	43 (31.2)	81 (58.7)^a^	18 (13.0)	108 (78.3) a	101 (73.2)^c^
≥40 years	98 (27.9)	169 (48.1)	38 (10.9)	237 (67.5)	189 (53.8)
Main area of practice					
Community A/N care	5 (17.2)^a^	15 (51.7) a	8 (27.6)^a^	21 (72.4)^b^	14 (48.3)^a^
Hospital ward	116 (32.1)	187 (51.8)	31 (8.6)^a^	257 (71.2)	225 (62.3)
Normal birth unit	8 (38.1)	14 (66.7)	6 (28.6)^a^	21 (100)	7 (33.3)
Outpatients Department	9 (22.0)	24 (58.5)	5 (12.2)	23 (56.1)	26 (63.4)
Education, management and other	3 (8.1)	10 (27.0)	6 (16.2)	23 (62.2)	18(48.6)
Type of post					
Midwife without leadership/specialist duties	126 (32.6)^b^	214 (55.3)^c^	46 (11.9)	279 (72.1)	235 (60.7)
Midwife with leadership/specialist duties	15 (15.2)	35 (35.4)	10 (10.1)	64 (64.6)	54 (54.5)
Content in practice					
One area	70 (28.8)	126 (51.9)	23 (9.5)	166 (68.3)	144 (59.3)
More than one area	69 (28.6)	123 (51.0)	33 (13.7)	176 (73.0)	145 (60.2)
Size maternity unit					
<2,500 births per year	77 (29.1)	96 (36.2)^c^	32 (12.1)	189 (71.3)	142 (53.6)^a^
≥2,500 births per year	64 (28.7)	154 (69.1)	24 (10.8)	156 (70.0)	148 (66.4)
Working hours					
Full time	87 (35.6)^a^	98 (44.5)^b^	18 (8.2) a	143 (65.0)^a^	164 (61.4)
Part‐time	53 (24.1)	151 (56.6)	38 (14.3)	200 (74.9)	125 (56.8)
Work distribution					
Daytime, weekdays only	8 (12.1)^b^	27 (40.9)	12 (18.2)	42 (63.6)	35 (53.0)
Shifts/weekends	133 (31.6)	223 (53.0)	44 (10.5)	301 (71.5)	254 (60.3)
Midwifery experience					
<10 years	46 (27.4)	99 (58.9)^a^	20 (11.9)	125 (74.4)	118 (70.2)^c^
≥10 years	93 (29.2)	149 (46.7)	35 (11.0)	218 (68.3)	170 (53.3)
Academic degree					
None	79 (31.3)	119 (47.2)	30 (11.9)	175 (69.4)	143 (56.7)
Any	141 (28.8)	131 (55.3)	26 (11.0)	170 (871.7)	147 (62.0)
Recent organization change					
No	73 (26.2)^a^	144 (51.6)	33 (11.9)	201 (72.0)	163 (58.4)
Yes	61 (35.7)	88 (51.5)	22 (12.9)	122 (71.3)	105 (61.4)

*p*‐values for each variable are indicated by superscript letter behind the first category of the variable.

a *p* < .05.

b *p* < .01.

c *p* < 0.001.

**Table 5:** Factors significantly associated with unfavourable working environment perception (means score < 2.5) in the Practice Environment Scales after adjustment for all significantly associated factors in Table 4

Characteristics	Quality of management	Resource adequacy	Midwife‐doctor relations	Opportunities for development	Midwifery foundation for care
	aOR (95% CI)	aOR (95% CI)	aOR (95% CI)	aOR (95% CI)	aOR (95% CI)
Main area of practice					
Community A/N care	2.56 (0.38–13.33)	3.75 (1.12–12.31)		1.43 (0.46–4.41)	
Hospital ward	4.55 (1.03–20.16)	2.88 (1.17–7.11)		1.19 (0.56–2.52)	
Normal birth unit	6.26 (1.10–35.51)	6.25 (1.66–23.55)		All midwives scored < 2.5	
Outpatients Department	4.99 (0.94–25.76)	5.39 (1.79–16.21)		0.69 (0.27–1.76)	
Education, management and other	1	1		1	
Type of post					
Without leadership/specialist duties	3.00 (1.45–6.19)	2.29 (1.28–4.10)			
With leadership/specialist duties	1	1			
Size maternity unit					
<2,500 births per year		1			1
≥2,500 births per year		4.50 (2.99–6.78)			1.63 (1.11–2.37)
Working hours					
Part‐time			1.87 (1.00–3.52)	1.53 (1.01–2.31)	
Full time			1	1	
Midwifery experience					
<10 years					2.01 (1.34–2.37)
≥10 years					1
Recent organization change					
No	1				
Yes	1.94 (1.26–2.97)				

**Table 6:** Themes and sub‐themes

Overall theme	The challenge of being a midwife in the 21st century			
Themes	Lack of resources	Insufficient support	Staying in midwifery	Lack of influence
Subthemes	Unable to provide woman‐centred quality care	A desire for recognition by midwifery leader	Requiring professional development	Powerless in a constantly changing work environment
	Fear of adverse events	Midwifery leader needs to be midwives’ advocate	The strain of shift work	Ruled by the medical model of care

Compared with midwives working in management, research and development or with special duties, midwives without leadership and special duties rated *Quality of management* and *Resource adequacy* significantly more often unfavourable, adjusted Odds Ratio (aOR) 3.00 (95% CI: 1.45–6.19) and aOR 2.29 (95% CI: 1.28–4.10), respectively. In addition, working at a large maternity unit was associated with an unfavourable evaluation of *Resource adequacy* (aOR 4.50, 95% CI: 2.99–6.78) and a *Midwifery foundation for care* (aOR 1.63, 95% CI: 1.11–2.37). A recent reorganization of the workplace was only significantly associated with an unfavourable rating of *Quality of management,* aOR 1.94, (95% CI: 1.26–2.97). Working part‐time was only just associated with the factors *Midwife‐doctor relations* and *Opportunities for development* (Table 5). While an academic degree, working shifts and weekends and working in more than one area were not associated with an unfavourable evaluation of the practice environment in the multivariate regression analyses (crude data in Table 4, adjusted data not shown).

### 5.2 Qualitative results

A total of 174 midwives contributed with open‐ended answers. Most comments concerned an unfavourable description and critical evaluation of the working environment. Content analysis revealed four main themes: (a) Lack of resources; (b) Insufficient support; (c) Staying in midwifery; and (d) Lack of influence. This was interpreted into one overall theme: The challenge of being a midwife in the 21st century. The themes and subthemes are presented in Table 6 and described below.

### 5.3 Lack of resources

Comments regarding this theme were most prominent. The two subthemes found in this theme were as follows: *Unable to provide woman‐centred quality care* and *Fear of adverse events*.

Not being able to provide adequate care was linked to a demanding workload and insufficient staffing. Midwives reported spending inappropriately much time on electronic documentation. They mentioned the lack of supporting personnel, for example to provide food, take blood test, clean labour rooms and order equipment:

We are busy, and the demands are increasing. Nobody is happy because we cannot do our jobs properly.

The midwives expressed a desire to provide woman‐centred care. They wanted to focus on normal birth and offer continuity of care and spent more time with the women:

Want to be able to offer women one‐to‐one care and not this birth factory.

Inadequate staffing led midwives to be afraid of adverse events. Several had experienced situations where they felt left alone without receiving the help they required/asked for in time. This made some of the midwives anxious on duty:

Our workload is increasing without the added resources. This is stressful and it does not feel safe. I do not want to end up killing someone.

### 5.4 Insufficient support

All the comments in this theme were about insufficient support from first‐line midwifery leaders. Midwives pointed out that midwifery leaders had a responsibility towards midwives who were in contact with women concerning the work they do. In addition, midwives expressed the need for a midwifery leader who defended their interests higher up in the organization. This resulted in two subthemes: *A desire for recognition by midwifery leader* and *Midwifery leader to be midwives’ advocate*.

Midwives expressed a need for their leaders to notice how well they did their job, recognizing their midwifery skills and great efforts;

I want better leaders and for them to see me and give me positive feedback.

The lack of support from the midwifery leaders on an organizational level was an issue. Midwives wanted someone who would be their advocate higher up in the organization. They wanted leaders who were loyal to midwives at grass root level and not upwards in the hierarchy:

The midwifery leaders are too timid. They are more loyal to their leaders above them compared to doctors… They have less power and influence than doctors.

### 5.5 Staying in midwifery

Midwives wrote about the requirements for and obstacles to staying in midwifery. These formed the two subthemes in this theme: *Requiring professional development* and *The strain of shift work*. It was common for the midwives to express a lack of opportunities for professional development. Midwives worried at how they should keep up to date with new developments in the field of midwifery and acquire new research‐based knowledge. They wanted to be able to attend courses but also to discuss cases with colleagues:

I want time to keep myself updated and I want time to discuss with my colleagues!

Many of the midwives in this sample found shift work hard and wanted more influence on their actual working hours. They expressed that it was not possible to work full time due to workload and irregular hours. They doubted they could work in this way until retirement:

I don't think I can last the professional life like it is today.

### 5.6 Lack of influence

The comments in this theme concerned the changes in the work environment, organization and model/culture of care. The changes were presented as ‘taking place’ without any or little involvement from the midwives. Two subthemes were identified in this theme: *Powerless in a constantly changing work environment* and *Ruled by the medical model*.

Approximately half of those replying to the open‐ended questions had experienced a recent reorganization of their workplace environment and most said they were not involved in the process. A reorganization led to new tasks and responsibilities or being moved to a different ward. Midwives had no power in the decision‐making process:

The hospital closed one ward during the summer, and we were moved to another one without much notice. We had no influence and it did not feel safe.

In addition to changes in the work environment, midwives described changes in the content of their work. These changes include more doctors involved in more births. They described an increased focus on pathology leading to more interventions. The medical model of care seemed to rule through procedures resulting in less autonomy of the midwife. For example, procedures indicating when to use continuous foetal monitoring or which technique to use for supporting the perineum in the second stage. As one midwife wrote:

The medical model influences our job and the focus on pathology makes us obstetrical nurses; just the doctors assistant.

All four themes were interpreted into an overall theme that describes the essence: *The challenge of being a midwife in the 21st century*.

### 5.7 Joint interpretation of the results

There was considerable overlap between the content of the subscales (factors) and the themes of the qualitative analyses. The PES seemed to have captured very well what midwives in the free text expressed as their concern for lack of adequate resources. The qualitative findings added midwives’ fear of adverse events related to this. Most midwives rated the subscale *Midwifery foundation for care* unfavourable. This agrees with the subtheme *Ruled by the medical model of care*. Both the quantitative and qualitative results showed that midwives perceived they need more opportunities for professional development. There are no items in the PES to match the qualitative finding that shift work posed a threat to midwives staying in midwifery. The favourable rating of the PES subscale *Quality of management* by most midwives is in contrast to the qualitative findings described in the theme *Insufficient support* and *Lack of influence* with the subtheme *Powerless in a constantly changing work environment*. However, the logistic regression analyses showed that midwives without leadership or specialist duties and midwives in the normal birth unit and those who had recently experienced a reorganization of their work environment were much more likely to rate the *Quality of management* unfavourable.

## 6 DISCUSSION

Our study showed that midwives in Norway found it a challenge to be a midwife in the 21st century. Both quantitative and qualitative data showed that midwives struggled with lack of resources, perceived they worked in a medical model of care, experienced insufficient support from their midwifery leaders and wanted more opportunities for professional development. Qualitative results added that midwives considered shift work as an obstacle to staying in midwifery.

### 6.1 Providing woman‐centred high‐quality midwifery care

The midwives in our study did not perceive their care was based on a midwifery foundation. Instead, they expressed being ruled by the medical model of care. A midwifery model of care has been described as one where the midwife can build a reciprocal relationship with the woman, creates a birthing atmosphere which supports normality and uses midwifery knowledge and skills (Berg, Asta Olafsdottir, & Lundgren, 2012). Caseload midwifery care appears to allow midwives to practice woman‐centred continuity of care across the continuum of pregnancy, labour and birth and the early parenting period (Fenwick, Sidebotham, et al., 2018). Continuity of care is rare in Norway. Most midwives practice in busy, fragmented maternity services. Midwives may experience continuity of care during the antenatal period and may meet the woman again postpartum. However, most women they care for during labour they will have never met before. As most births take place in hospital, doctors have enormous influence over the care provided by midwives through procedures, their presence and authority. Our findings show that midwives experienced excessive focus on risks and use of interventions as obstacles for providing woman‐centred high‐quality care based on a midwifery foundation.

Quality problems in health care have been classified into three categories: overuse, underuse and misuse of care (Jones, Hamilton, & Murry, 2015). In our study, overuse was identified as the use of unnecessary interventions. Misuse was described as the same high‐risk approach to all women. Underuse was exemplified by insufficient time spent with birthing women. The seriousness of inadequate resources, midwife shortage and/or poor staff mix was expressed through the fear of adverse events. There is ample evidence that being able to provide good quality care has a positive effect on the emotional and professional well‐being of midwives, while not being able to do so makes midwives want to leave their profession (Pezaro et al., 2016; Pugh et al., 2013). A recent Australian study showed that midwives providing continuity of midwifery care reported lower levels of burnout, depression and anxiety and higher levels of professional identity and autonomy compared with midwives working in fragmented care (Fenwick, Sidebotham, et al., 2018). Providing good care for women is good for midwives.

While midwives may not have liked the medical model of care where they work, most midwives rated their relationship with doctors as favourable. As in Sweden, where the same observation was made, midwives and doctors have a history of collaborating well together while at the same time midwives report that their own professional role is eroding (Hildingsson & Fenwick, 2015; Larsson, Aldegarmann, & Aarts, 2009).

### 6.2 Staying in midwifery

In Norway, midwives working in hospitals are obliged to work shifts and weekends. A three‐shift system of day, evening and night shift of 8–10 hr is common, while some places have 12‐hr shifts. Retirement age is 65 years, for those working in hospitals. A previous publication on this dataset showed that midwives in Norway with increasing age move to workplaces without shifts and weekends such as community antenatal care and the outpatient department (Henriksen & Lukasse, 2016). This represents a considerable drain of expertise away from care for childbearing women and their babies admitted to hospital. Critical losses in the midwifery workforce led researchers in Western Australia to investigate why midwives left their post or profession (Pugh et al., 2013). Midwives cited inflexible duty rostering contributing to a work–life imbalance as a reason for leaving while the opportunity of professional development was identified as an issue supporting staying (Pugh et al., 2013).

In contrast to some other countries and other professions, Norway has no statutory rules ensuring midwives participate in continuing professional development after completing midwifery education (Gray, Rowe, & Barnes, 2016). Thus, employers are not obliged to provide midwives with the opportunity for further professional development and education. The law states that health professionals are responsible to keep up to date with changes in their professional practice (Helsepersonelloven, 2019). Most workplaces in Norway have regular obligatory courses for midwives which focus on training skills and practicing teamwork in relation to acute serious obstetric events. In addition, they distribute information to their employees via emails or at existing meetings. Thus, they aide midwives in keeping up to date. Midwives however expressed the desire to attend courses, conferences and have more time to discuss practice with colleagues. Midwives mentioned this as part of their evaluation of their work environment. Thus, the comments appear to imply the expectation that their employer should support them in this. Remuneration of expenses for attendance fees and/or paid leave to attend external courses/conferences has become rare.

### 6.3 Midwifery leadership

Of all the participants in our study, about a third had an unfavourable rating of their midwife leaders. There were no specific open questions on midwifery leadership/management. However, midwives did report insufficient support from their ‘own midwife leader.’ A study of accounts of midwives, asked to characterize ‘good’ leadership in midwifery, identified that besides ‘skilled competence’ the extent of their ‘emotional intelligence’ made a leader ‘good’ (Byrom & Downe, 2010). Similarly, a systematic review of leadership styles and outcome patterns for the nursing workforce and their environments identified that leadership styles focused on people and relationships, as opposed to tasks, were associated with higher job satisfaction (Cummings et al., 2010). Our findings, in agreement with the literature, emphasize the importance of midwife leaders. Not in the least as feeling supported and valued by managers was part of the reason ‘why midwives stayed in the profession’ in a study by Curtis, Ball, and Kirkham (2006).

### 6.4 The Practice Environment Scale

The PES:Midwives, a modified version of the PES, with only 20 items in 4 subscales was not available when our current study was performed (Pallant et al., 2016). As in the study by Pallant et al. (2016) and Hildingsson and Fenwick (2015), our participants answered 30 questions. An analysis of all their answers seemed both ethically correct and of interest scientifically. Midwifery and midwives’ practice environment are quite different in Norway compared with New Zealand. A five‐factor solution, as in the original PES (Lake, 2002), suited our data better than the proposed 4‐factor solution in the PES:Midwives from New Zealand (Pallant et al., 2016). The extra factor in our 5‐factor solution was *Midwifery foundation for care*. This factor has less items but is similar to the original developers’ factor *Foundation for quality care* (Lake, 2002). Hildingsson & Fenwick investigating Swedish midwives’ perceptions of their practice environment identified this factor as *Midwifery Foundation of Quality Care* (Hildingsson & Fenwick, 2015).

Interestingly, in the Norwegian sample, the items that originally loaded under influencing hospital affairs did not load at all. Yet, our qualitative results show that midwives did not feel they participated in decision‐making about their work environment/hospital affairs. They mentioned constant changes they were unable to influence. Our findings suggest the inclusion of some questions in the PES on reorganizations: physical environment, leadership, personnel, responsibilities, shifts and rotation, new technical equipment and new computer programs. There is evidence that design and layout of hospital wards and labour rooms; and support systems such as personnel to provide food, take bloods, clean beds and rooms; and computerized patient information systems greatly influence how midwives feel about their work (Darbyshire, 2004; Hammond, Homer, & Foureur, 2017).

### 6.5 Limitations

A limitation of the study is the poor response rate, a result of observing total anonymity prohibiting reminders could be sent. However, our study sample included about 20% of all midwives in Norway who worked in a hospital setting. A bias towards more dissatisfied midwives participating is possible and caution is needed regarding the generalizability/transferability of the results. However, compared with the usual few participants in qualitative studies, our study included many midwives who provided extensive answers to the open‐ended questions.

## 7 CONCLUSION

Our mixed methods study shows that the adapted PES can be used to measure midwives´ perception of their workplace environment. Based on the qualitative findings and the literature, we suggest some questions are added to capture midwives’ perceptions of changes in organizational structure, support systems and design and layout of their work environment, as well as issues regarding shifts, use of technology and computerization. Norwegian midwives perceived being a midwife in the 21st century as challenging. To prevent experienced midwives from leaving midwifery care in obstetric units requires a more flexible approach to shifts and the duty roster. Good midwifery leaders are pivotal to the professional and emotional well‐being of midwives. To encourage midwives to stay in midwifery, continuous professional education is needed as well as the opportunity to practice woman‐centred high‐quality care.

## CONFLICT OF INTEREST

The authors do not have any conflict of interest to declare.

## AUTHOR CONTRIBUTION

ML and LH: conceptualization and design, or acquisition of data, or analysis and interpretation of data; manuscript drafting and critical revision for intellectual content. All authors approved the final version of the manuscript and agreed to be accountable for all aspects of the work in ensuring that questions related to the accuracy or integrity of any part of the work are appropriately investigated and resolved.

## RESEARCH ETHICS COMMITTEE APPROVAL STATEMENT

The study was submitted to the Medical and Health Research Ethics board who deemed their approval not required and the study not within their scope. The Norwegian Social Science Services (NSD) approved the study. Lists with names and addresses were handled confidentially by the printers and destroyed after posting. Midwives were informed that the returning of a completed questionnaire was considered to be their consent to participate.

## PATIENT CONSENT STATEMENT

No patients were included in this study.
