# Supplementary material for: Norwegian midwives' perceptions of their practice environment: A mixed methods study
Source: Nurs Open. 2019 Aug 6;6(4):1559–70. doi: 10.1002/nop2.358 (PMC6805784; doi:10.1002/nop2.358)
Supplement: Supplementary file 1 [file NOP2-6-1559-s001.docx]

Table S1: Open ended questions

| If you have been part of a re-organization, tell how you experienced this. |
| --- |
| How do you think the maternity care should change? How would you like to work? |
| Do you think your work environment has changed over the last years? If yes, how? |
| Have you been in a situation that have made you consider leaving your job? In that case, what kind of situation? |
| Have you been in a situation at work where you felt scared? Tell us what happened and what you felt. Were you offered any help to process the situation if needed? |
| Do you have any suggestion related to how the environment can improve for the midwives to be able to work longer? |
| Are there any other points or ideas you want to share? |
