# Supplementary material for: Norwegian midwives' perceptions of their practice environment: A mixed methods study
Source: Nurs Open. 2019 Aug 6;6(4):1559–70. doi: 10.1002/nop2.358 (PMC6805784; doi:10.1002/nop2.358)
Supplement: Supplementary file 2 [file NOP2-6-1559-s002.docx]

Table S2. Example of the analysis process with condensed meaning units, codes, categories, subthemes and themes

| Condensed meaning unit | Code | Category | Subtheme | Theme |
| --- | --- | --- | --- | --- |
| Want to provide continuity of care and spend more time with the women  We need more staff  The demands are increasing. We can not do our job properly  There is an increasing workload with no added recourses | Continuity of care  One-to-one care  Not enough staff  Increasing demands  Increasing workload  Not enough resources | Women-centred care  Resources  Adequate care  Resources | *Unable to provide woman-centred quality care* | **Lack of resources** |
| Been scared in situations because of really busy shifts. | Have been scared  Busy shifts | Fear  Resources | *Fear of adverse events* |  |
| The working environment is changing all the time. New tasks are added.  We had a difficult process when one of the labor ward was closed down, no influence | Constantly changing work environment  No influence when working environment is changing | Working environment  Not being involved  Working environment | *Powerless in a constantly changing work environment* | **Lack of influence** |
| More and different tasks, more stress more focus on pathology and risks  The medical model influences our job | Constantly changing work tasks  An increasing risk-based approach  Medical model of care | A risk-based approach  A risk-based approach  Medical model | *Ruled by the medical model* |  |
